# Activin subfamily peptides predict chronological age in humans

**DOI:** 10.14814/phy2.13823

**Published:** 2018-09-03

**Authors:** Lady V. Barrios‐Silva, Mack Parnell, Zahida B. Shinwari, Ghulam A. Chaudhary, Thanasis Xenofontos, Angel van Bekhoven, Simon McArthur, Bradley T. Elliott

**Affiliations:** ^1^ Translational Physiology Research Group Faculty of Science and Technology University of Westminster London United Kingdom; ^2^ Engineering & Applied Science Hogeschool Rotterdam Rotterdam Netherlands; ^3^ Institute of Dentistry Queen Mary University of London London United Kingdom

**Keywords:** Aging, frailty, GDF11, GDF8, myostatin

## Abstract

Loss of muscle mass and function are a well‐defined aspect of human aging from the 3rd decade of life, which result in reduced independence and increased mortality. The activin family of peptides contains several endocrine factors (activin A, myostatin, growth and differentiation factor 11 [GDF11]) that may play roles in changes in muscle mass and the aging process, however, it may be simplistic to consider aging as a result of a single peptides changes. Thus, we aimed to examine changes in activin family members across a cohort of healthy individuals of various ages, hypothesizing that these would aid predictive models of age and functional measures of age. Healthy participants (*n* = 88) were recruited and resting metabolic rate, body composition, grip strength, walking speed, and circulating plasma concentrations of myostatin (total and free), activin A, follistatin‐like binding protein (FLRG), and GDF11 quantified. Simple regressions between circulating factors and chronological age, grip strength, and walking speed were examined. Multiple stepwise regressions for age, grip strength, and walking speed are also reported. Age negatively correlated with total myostatin (*P* = 0.032, *r* ^2^ = 0.053), grip strength positively with activin A (*P* = 0.046, *r* ^2^ = 0.048), whereas walking speed showed no simple regression relationships. Stepwise regressions suggested a role of total myostatin and activin A in models of age, whereas GDF11 contributed to the model of grip strength. Here we suggest a role for myostatin, activin A, and GDF11 in normal human aging that mirrors animal studies to date. Further interventional studies are required to elicitate the physiological role of these changes in the normal human aging process, and indeed if offsetting these changes can promote successful aging.

## Introduction

The aging process is inherent to the human condition, and involves a loss of muscle mass, muscle function, and a generalized increase in frailty. Loss of muscle mass and function occurs at a predictable rate throughout adulthood, with evidence of muscle mass loss from 30 years of age (Kallman et al. 1990; Mitchell et al. 2012; Steiber 2016). At its extreme, sarcopenia is seen in 5–13% of people between 60 and 70 years of age, which increased to 50% of those >80 years of age (Metter et al. 2002). Importantly, Western society continues to show an aging shift; thus the proportion of older individuals with reduced muscle function and sarcopenia is predicted to grow, increasing the load on national healthcare systems (Harper 2014; Bloom et al. 2015).

Myostatin, also known as growth and differentiation factor 8 (GDF8; McPherron et al. 1997) is a member of the transforming growth factor beta (TGF*β*, activin clade; Hinck et al. 2016) superfamily. Released mainly by skeletal muscle, it acts to negatively regulate muscle mass through its antianabolic and procatabolic effect directly on myocytes (Elliott et al. 2012). Myostatin in plasma can be found in three states, free (unbound), bound to its own propeptide, or bound to one of several inhibitory proteins, with total myostatin thus representing free plus bound myostatin. Propeptide binding prevents myostatins’ atrophic actions (Thies et al. 2001; Jiang et al. 2004), as does binding to the inhibitory peptides such as follistatin or follistatin‐related gene protein (FLRG; Hill et al. 2002; Amthor et al. 2004; Gilson et al. 2009). Myostatins’ role as an endocrine hormone was first suggested in humans, where a negative correlation between muscle mass and myostatin peptide concentration in serum was noted across healthy and cachexic HIV patients (Gonzalez‐Cadavid et al. 1998). This endocrine role was reinforced by Zimmers et al. (2002) who inserted Chinese hamster ovary (CHO) tumors peripherally in mice, modified to produce and secrete myostatin. Myostatin secreting tumor mice showed an increase in serum myostatin concentration and loss of global muscle mass, whereas CHO nonproducing tumors did not.

The role of myostatin in the human aging process has not been well‐defined in the literature to date. Schulte and Yarasheski (2001) first demonstrated that free myostatin was elevated in serum of older frail woman and inversely correlated with muscle mass. Furthermore, increased levels of the free fraction of serum myostatin have been reported in a cohort of 60‐ to 75‐year‐old participants relative to a 19‐ to 35‐year‐old cohort (Yarasheski et al. 2002). Conversely, no significant difference between serum myostatin was seen between young and sarcopenic elderly men, nor were any differences between ages noted in the myostatin binding proteins follistatin and FLRG (Ratkevicius et al. 2011). These studies have used between‐participant models, examining discrete cohorts of young/old participants. If there is a change in myostatin with age, it would be reasonable to hypothesize that is would be linear and positive, reflecting the changes in muscle mass and strength that occur during aging.

Other members of the activin subfamily with structural, and potentially functional, similarity to myostatin exist, including activin A and growth and differentiation factor 11 (GDF11; Hinck et al. 2016). Activin A has received much attention with regards to aging, and tends to increase in later life (Baccarelli et al. 2001; Ebert et al. 2006). Much recent attention has been placed on GDF11 in the aging process. Indeed, the concentration of GDF11 in middle‐aged mice is predictive of all‐cause mortality with aging (Zhou et al. 2016). In older mice, GDF11 is reduced relative to younger, and exogenously supplied GDF11 restores muscle function toward “younger” values (Sinha et al. 2014), whereas maintenance of muscular function with aging in humans is associated with elevated GDF11 concentrations (Elliott et al. 2017).

A better understanding of the change in these activin family peptide appears needed, both to begin to gain insight into their role in aging, and also to develop quantifiable biomarkers of biological aging. Thus, the aim of this work was to describe changes in myostatin and closely related activin‐related peptides with age, by recruiting a large cohort of declared healthy individuals with a cross‐sectional representation of ages. It was hypothesized that activin A and total myostatin would correlate positively with age, whereas FLRG and GDF11 would negatively correlate.

## Materials and Methods

### Ethical approval

Ethical approval was obtained by the Faculty of Science and Technology Research Ethics sub‐Committee, University of Westminster (#VRE1516‐0084). All work herein conforms to the standards set by the Declaration of Helsinki. Written informed consent was obtained from all participants prior to participation.

### Participant descriptors

Declared healthy individuals (range 18–68 years of age, *N* = 88, 39 males) were recruited for this study. Participant sex was self‐declared. Inclusion criteria was 18–80 years of age and clinically stable, with no known cardiovascular, respiratory, metabolic, or coagulation disorders, no disorders of muscle mass, not a regular smoker or currently taking any prescription medication (excluding hormonal contraceptive methods). All participants were required to have a BMI of 18–30 kg/m^2^.

### Experimental design

Each participant was asked to refrain from structured exercise for 24 h prior to testing, and fast (nothing but water) for 12 h prior to testing, therefore presenting for testing in a fasted, rested state. All participants were tested between 09:00 and 12:00. The order of testing was as follows; resting metabolic rate, body composition, venous blood sample, grip strength, and 6‐min walk test (6MWT). Order was strictly adhered to, and completed within 90‐min per participant.

### Resting metabolic rate

Resting metabolic rate (kj/day) was determined by the use of indirect calorimetry. Briefly, participants lay supine on a bed in a darkened, thermoneutral environment with a mask covering the nose and mouth. Continuous gas exchange was monitored for 40 min (Cortex 3B, Biophysik, Germany), with the final 10 min of data presented here, to ensure participants metabolic rate had plateaued. Immediately on conclusion of the testing, resting heart rate, and blood pressure were recorded while the participant was still in a supine position.

### Body composition

Height was measured by stadiometer (Seca, Germany) to 0.1 cm, whereas weight was taken on a calibrated set of scales to 0.1 kg, with the same stadiometer and scales used for all participants. Body composition was measured by air volume displacement (Bod‐Pod, Life Measurement, USA. All measures utilized the same equipment. For testing, participants were asked to remove all jewelery and wear minimal, form‐fitting clothing and a swim‐cap. Participants were asked to breathe normally and avoid excessive movement during testing.

### Grip strength

As grip strength is indicated as a marker of muscle function in aging populations (Cruz‐Jentoft et al. 2010), assessment of grip strength was executed in a standard manner using a hand‐grip dynamometer (TKK501, Takei Scientific Instruments, Japan) with the participant standing with the arm straight and palm facing medially. Grip was measured three times per participant, in the dominant hand, with at least 1‐min break between measurements. One hand‐grip dynamometer was used for all measures. The mean of three measures is reported here.

### Venous plasma

Venous blood samples were drawn in a standard manner from a convenient vein of the cubital fossa into 6 mL lithium heparin tubes. Samples were centrifuged immediately (10 min, 4470 *g*, 4°C), with resultant plasma aliquoted and frozen (−80°C) for further analysis of peptide concentrations (described below).

### Six‐minute walk test (6MWT)

A 6MWT was completed in a standard manner (Holland et al. 2014). Briefly, participants were instructed to walk at a self‐paced velocity, with the verbal instruction to walk as far as possible, but not to run or jog. Testing was done indoors, on a 10 m linear track marked in an institutional hallway, with distance travelled quantified to the nearest meter. All participants were tested on the same marked track.

### ELISA

Aliquots of plasma were analyzed for total myostatin, free myostatin (both DGDF80, R&D Systems, UK), FLRG (DFLRG0, R&D Systems, UK), activin A (DAC00B, R&D Systems, UK), FLRG (DFLRG0, R&D Systems, UK), and GDF11 (DY1958, R&D Systems, UK) were analyzed according to manufactures instructions. Total myostatin was measured by the acidification release method. Briefly, 100 *μ*L plasma was treated with 50 *μ*L 1N HCl, vortexed and incubated at room temperature for 10 min then neutralized with 50 *μ*L 1.2 N NaOH and further diluted in 200 *μ*L manufacturer provided diluent for a final dilution of 1:4, similar to previously validated methods (Lakshman et al. 2009). Plasma samples for free myostatin were diluted 1:4 in identical diluent, without acidification or neutralized treatment. All samples and standard were analyzed in triplicate. Of the 88 participants, 56 GDF11 results were either below limit of detection of our assay (31.3 pg/mL) or otherwise undetectable, and are thus not reported here.

**Figure 1:**
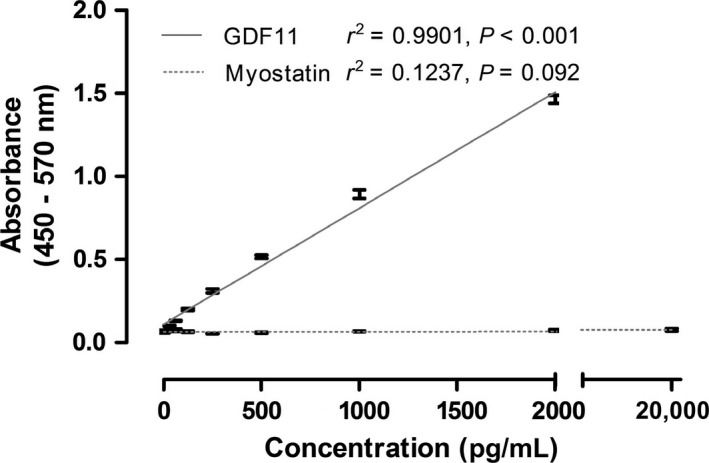
GDF11 ELISA antibody does not detect recombinant myostatin in a physiological or supra‐physiological range. Concentration of GDF11 (0–2000 pg/mL) and myostatin (0–20,000 pg/mL) as a function of absorbance (450–570 nm). Samples run in triplicate and reported here as mean with standard error. Solid line indicates linear regression for GDF11, dashed line for myostatin.

As GDF11 has been previously reported to have a high structural similarity to myostatin/GDF8 (Poggioli et al. 2016), we also directly compared the GDF11 ELISA used here against recombinant myostatin in a physiological (0–2000 pg/mL) and supra‐physiological (20,000 pg/mL) range, with no cross reactivity reported (Fig. 1).

### Statistics

All figures were generated in GraphPad Prism (Version 5, GraphPad), and statistical analysis was performed using SPSS (version 23, IBM). Pearson's correlation was used out to examine possible associations between collected endocrine variables and a) age (years), b) grip strength (kg) and c) 6‐min walk distance (m). A *P*‐value of less than 0.05 was considered significant throughout. Automatic linear modeling was used for forward stepwise regression with an endpoint when Akaike information criterion, conservative (AICC) was minimized. Predictor importance of generated models were calculated as the residual sum of squares with the variable removed from the model, normalized to a sum of 1 to indicate relative value of individual factors to the total model. Outlines were removed if > 3 SD from mean, as recommended by Yang (2013).

## Results

### Activin factors predict age and grip strength

**Figure 2:**
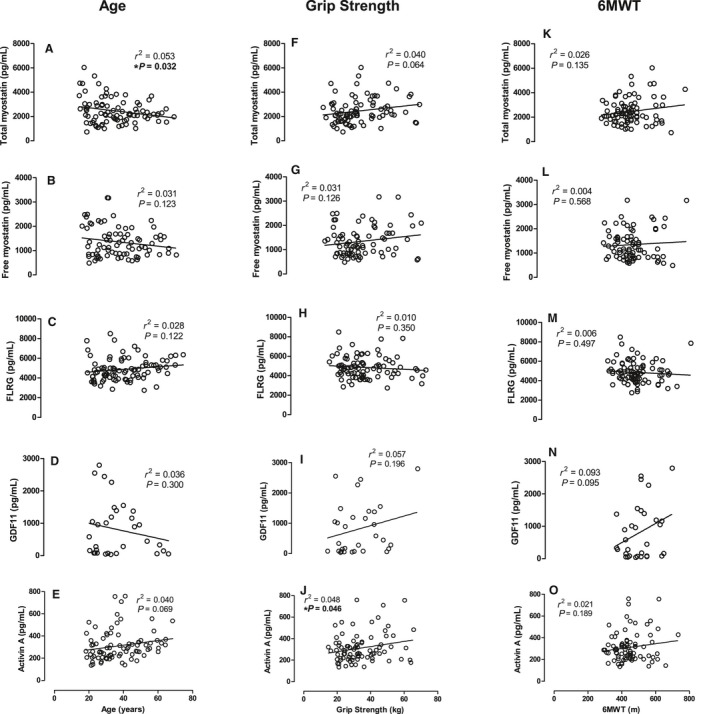
Peptide concentration (pg/mL) as a function of age (y*ears), grip strength (kg) and 6‐*min walk performance (m). (A–E) Age (years*), (F–J) Grip strength, (K–O) 6‐*min walk distance covered (m), as a function of concentration of total myostatin (top row), free myostatin (second row), FLRG (middle row), GDF11 (forth row), and activin A (bottom row). All concentrations in pg/mL.

To examine the relationship between myostatin peptide family members and chronological age, the concentration of each peptide was expressed as a function of age (years). Total myostatin showed a negative linear correlation with age (*P* = 0.032, *r* ^2^ = 0.053, Fig. 2A), whereas free myostatin did not (*P* = 0.123, *r* ^2^ = 0.031, Fig. 2B). FLRG did not show a correlation with age (*P* = 0.122, *r* ^2^ = 0.028, Fig. 2C), nor did GDF11 (*P* = 0.300, *r* ^2^ = 0.036, Fig. 2E) or activin A (*P* = 0.087, *r* ^2^ = 0.040, Fig. 2E, Table 1).

**Table 1:** Activin family members (pg/mL) by age (years). Data are given as mean (standard error)

Age (years)	Total myostatin	Free myostatin	FLRG	Activin A	GDF11
18–29	2758.5 (270.6)	1386.4 (149.2)	4563.8 (202.2)	261.2 (19.5)	774.8 (243.6)
30–39	2450.5 (190.3)	1508.8 (146.3)	4976.4 (269.7)	352.7 (33.7)	1176.7 (289.4)
40–49	2137.5 (159.1)	1165.7 (104.6)	4883.0 (261.5)	298.4 (32.1)	963.8 (155.8)
50–59	2496.1 (198.5)	1332.2 (165.7)	4929.4 (231.3)	351.3 (32.8)	177.4 (80.8)
60<	2035.9 (171.7)	1240.3 (143.4)	5720.1 (303.8)	351.5 (40.1)	52.5 (—)^a^

a *n* = 1 participant over 60 years of age participant had GDF11 concentrations above the limits of detection, thus no error is reported on this value.

With regards to functional measures of aging, activin A shows a positive linear correlation with grip strength (*P* = 0.046, *r* ^2^ = 0.048, Fig. 2J). No other endocrine factors correlated with grip strength, and no endocrine factors correlated with 6‐min walk distance covered (Fig. 2K–O, Tables 2 and 3).

**Table 2:** Activin family members (pg/mL) by grip strength (kg), binned by quartiles

Grip strength (quartiles)	Total myostatin	Free myostatin	FLRG	Activin A	GDF11
<24	2281.8 (194.4)	1444.7 (130.5)	5138.3 (525.3)	298.7 (23.6)	320.3 (33.6)
25–49	1849.6 (147.9)	985.0 (71.9)	4707.2 (232.9)	265.6 (22.5)	280.8 (18.2)
50–74	2940.8 (258.9)	1350.2 (131.5)	4713.8 (207.4)	308.3 (29.2)	400.0 (96.2)
75<	2741.1 (211.3)	1590.0 (169.8)	4837.6 (275.5)	372.1 (36.2)	417.7 (92.4)

Data are given as mean (SE).

**Table 3:** Activin family members (pg/mL) by 6 min walk distance (m), binned by quartiles

Grip strength (quartiles)	Total myostatin	Free myostatin	FLRG	Activin A	GDF11
<24	2288.5 (154.0)	1380.3 (98.9)	5236.8 (259.8)	287.5 (20.0)	356.8 (84.8)
25–49	2160.9 (178.2)	1291.9 (160.9)	4876.0 (229.5)	296.0 (23.7)	325.5 (44.5)
50–74	2666.2 (218.5)	1296.2 (101.5)	4565.7 (208.5)	335.0 (35.7)	325.4 (27.0)
75<	2738.0 (319.8)	1420.4 (184.6)	4712.9 (259.3)	331.8 (36.3)	428.1 (106.2)

Data are given as mean (SE).

### Activin peptides aid prediction of age and frailty

While only total myostatin significantly correlated with age, albeit weakly with a small coefficient of determination, we next wished to examine the contribution of traditionally relevant makers of aging (body composition and cardiovascular health) and the activin family of peptides to chronological aging and aging‐associated markers of frailty, using a linear stepwise regression model.

**Figure 3:**
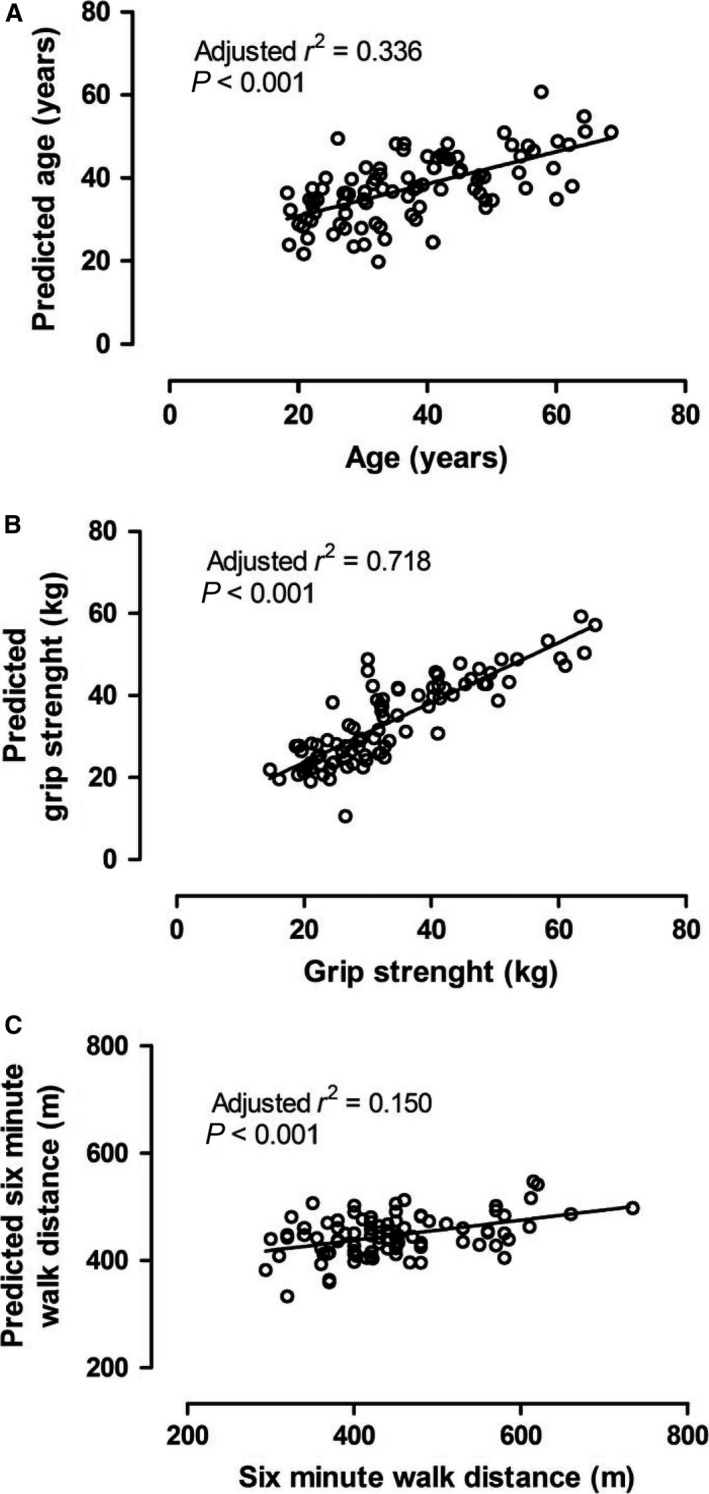
Prediction of aging factors as a function of actual factor. (A) Predicted age (years) as a function of age (years). (B) Predicted grip (kg) as a function of grip (kg). (C) Predicted 6MWT distance (m) as a function of 6MWT distance (m).

Systolic blood pressure was the primary predictor of chronological age from the variables we collected here (*r* ^2^ = 0.129, predictor importance = 0.365, *P* < 0.001), with a moderately strong model of age being formed by systolic blood pressure, total myostatin, resting metabolic rate, resting heart rate, activin A, fat mass, and 6‐min walk distance (adjusted *r* ^2^ = 0.336, predictor importance = 1.0, *P* < 0.001; Table 4) with no other variables further adding to this model (Fig. 3A). Grip strength, as one of the two measures of function recorded, was best predicted by fat‐free mass (*r* ^2^ = 0.607, predictor importance = 0.560, *P* < 0.001), and made a very strong model with the addition of sex, 6‐min walk distance and GDF11 (adjusted *r* ^2^ = 0.718, predictor importance = 1, *P* = 0.009, Table 5), with no other variables adding to this model (Fig. 3B). Finally, 6‐min walk distance was best predicted by grip strength (*r* ^2^ = 0.275, predictor importance = 0.346, *P* = 0.008). A weak model of prediction of 6‐min walk distance (m) was formed by grip strength, diastolic blood pressure, age, and BMI (adjusted *r* ^2^ = 0.150, predictor importance = 1, *P* < 0.001) with no other variables adding to the strength of this model (Fig. 3C, Table 6).

**Table 4:** Forward stepwise regression for age (years)

Model	Variable	AICC	Predictor importance
1	Systolic blood pressure (mmHg)	461.2	0.365
2	….. + Total myostatin (pg/mL)	454.2	0.170
3	….. + Resting metabolic rate (kj/day)	450.4	0.120
4	….. + Resting heart rate (beats/min)	448.3	0.118
5	….. + Activin A (pg/mL)	446.2	0.096
6	…. + Fat mass (kg)	444.2	0.071
7	…. + 6 min walk (m)	442.5	0.060

**Table 5:** Forward stepwise regression for grip strength (kg)

Model	Variable	AICC	Predictor importance
1	Fat free mass (kg)	369.7	0.560
2	…. + Sex	349.3	0.336
3	…. + 6 min walk (m)	343.0	0.080
4	…. + GDF11 (pg/mL)	342.9	0.024

**Table 6:** Forward stepwise regression for 6‐min walk (m)

Model	Variable	AICC	Predictor importance
1	Grip strength (kg)	801.9	0.346
2	…. + Diastolic blood pressure (mmHg)	800.3	0.227
3	…. + Age (years)	799.6	0.226
4	…. + BMI (kg/m^2^)	796.8	0.201

## Discussion

In line with our hypothesis, here we demonstrate that both total myostatin and activin A appear to independently predict age, along with traditionally recognized aging factors such as blood pressure and muscle mass. However, counter to our hypothesis, the total myostatin/age relationship was negative, not positive. This involvement of the activin group of peptides in the aging process is reinforced by our finding that total myostatin, activin and GDF11 fit into predictive regression models of both age and a functional change with age (grip strength), but possibly in a manner that we do not yet physiologically understand. Importantly, these factors fix complex, but not simple regressions, suggesting integrated models should be considered.

### Myostatin

Myostatin's central roles in the regulation of muscle mass have been previously described (Elliott et al. 2012). The role of aging in myostatin concentration has received prior attention, with mixed results. Prior cohort based approaches (younger compared to older) have previously been reported, with one suggesting an increase in plasma myostatin in older woman relative to younger (Yarasheski et al. 2002), a similarly designed examination showed no difference in plasma myostatin between a young and older cohort (Ratkevicius et al. 2011), and the third suggesting serum myostatin was decreased in older individuals relative to younger (Lakshman et al. 2009). To better examine this disparity, we selected a cross‐sectional approach. While we show a negative correlation between age and total myostatin, our results most closely mimic those of Ratkevicius et al. (2011) and Lakshman et al. (2009). Indeed, had we used a young/older cohort approach in our study, grouping our participants into younger (under 35 years of age, *n* = 44) and older (over 50 years of age, *n* = 17) our results would be in agreement with Ratkevicius et al. (2011), showing no difference between myostatin concentration between groups (young = 2631.4 (188.7), older = 2323.5 (143.2) pg/mL, unpaired t‐test, *P* = 0.351 [data not shown]).

While the role of total myostatin in the aging process remains unclear from this report, the well‐recognized role of myostatin in muscle size regulation (Elliott et al. 2012) makes it tempting to speculate that myostatin is playing a role in the aging process. Alternatively, it is equally plausible that the muscle mass decreases seen with increasing age are resulting in a reduced systemic concentration of total myostatin. While causation is not shown by this work, this correlative relationship is of interest to note and requires further investigation.

While pharmacological inhibition of myostatin is noted to result in increased muscle mass in mice (Whittemore et al. 2003), causative data in humans does not yet exist. Observational human data suggest that the K153R myostatin SNP variant associates with grip strength in an older female cohort (Seibert et al. 2001), and also associated with reaching 100 years of age (Garatachea et al. 2013). These data suggest that myostatin may still form a useful target for counteracting the loss of muscle function, and the subsequent health impact, with age.

### GDF11

Much recent attention has been placed on the role of GDF11 in aging and frailty. The concentration of circulating GDF11 in middle‐aged mice predicts life span (Zhou et al. 2016), whereas the provision of exogenous GDF11 in older mice restores age‐related decreases in grip strength and endurance performance in mice (Sinha et al. 2014). It has also been reported that that GDF11 and myostatin had similar, atrophic effects on myocytes in vitro and muscle cells in vivo, acting via the same receptor and SMAD‐dependent pathway (Egerman et al. 2015). Indeed, GDF11 has a highly similar sequence homology with myostatin (Hinck et al. 2016). It should be noted that myostatin in vivo circulates at much higher concentrations than GDF11 (we report here myostatin in the ng/mL range, while GDF111 is in the pg/mL range), potentially explaining this contradictory result. Indeed, Poggioli et al. (2016) conducted a careful analysis of antibody specificity with GDF11, and reported decreased GDF11 across multiple mammalian species with aging, in line with our results reported here. Finally, we do note that our assay shows specificity for GDF11 (Fig. 1), and does not interact with myostatin, a caution that others have previously raised (Poggioli et al. 2016).

We here further the results of others in small mammal models (Loffredo et al. 2013; Sinha et al. 2014; Poggioli et al. 2016), but for the first time in an aging human population. If the role of GDF11 in the human is maintained from these small animal models, then future work should target this aging‐associated decrease in GDF11 expression. An exciting hypothesis would be that restoration of GDF11 to “younger” levels would result in a restoration in muscle function and offset frailty, as has been suggested in mice (Sinha et al. 2014). Indeed, as we recently reported, successfully aging older individuals show higher expression of GDF11 than body mass‐matched control older individuals (Elliott et al. 2017), utilizing the method of measuring GDF11 that we report here. Besides this report, limited work has examined GDF11 and human aging. Further work targeting alterations in GDF11 in aging humans, either by environmental or pharmacological approaches, and subsequent changes in muscle function, is clearly needed.

### Activin A

Of the members of the activin clade of endocrine signaling peptides examined here, the role of activin A in aging is best understood. Indeed, Baccarelli et al. (2001) reported activin A did not change in healthy males and postmenopausal women before 60–70 years of age, using a similar cross‐sectional design as that used here. These results were mirrored by Ebert et al. (2006). A smaller longitudinal cohort suggested that activin A was increased over 10 years in males, but not females (Loria et al. 1998). Furthermore, serum from older women (43–47) show increased activin A relative to younger (19–38) women (Santoro et al. 1999). Our results mirror those previously reported (Baccarelli et al. 2001; Ebert et al. 2006) with no change in activin A in a healthy population of younger‐to‐older individuals.

### FLRG

FLRG is an inhibitor of myostatin, yet its effects on aging has received considerably less attention. We chose to examine it here to investigate the hypothesis that aging‐associated frailty may involve myostatin via a decrease in FLRG‐dependent inhibition. The results we present here suggest that FLRG is not involved in the aging process, with no alteration in chronological age or either marker of function (grip strength and walking speed), nor any involvement in our regression models. These findings are supported by Ratkevicius et al. (2011) who showed no change in FLRG, nor other myostatin inhibitors (follistatin or growth/differentiation factor‐associated serum protein‐1 [GASP‐1]), matching our results here.

## Conclusions

Here we report that the concentration of total myostatin tends to decrease as a function of age in otherwise healthy individuals, in addition total myostatin, activin A and GDF11 are predictive of chronological age and grip strength. As an inhibitor of myostatin, FLRG does not appear involved in the normal aging process. The decrease in total myostatin appears to be involved in the aging process, as it is the only factor that fits into both our simple and complex regression models of aging. The GDF11‐grip strength findings are exciting, as it mimics data seen previously in aging mice. We thus suggest that future research into modification of GDF11 and myostatin levels in the aging human are needed.

## Conflict of Interest

The authors declare that they have no competing interests.
